# Subtle Cerebellar Features in Relatives of Essential Tremor Cases

**DOI:** 10.3389/fneur.2020.00605

**Published:** 2020-07-17

**Authors:** Evan A. Hale, Ruby Hickman, Hollie Dowd, Deepti Varathan, Gina Liu, Elan D. Louis

**Affiliations:** ^1^Division of Movement Disorders, Department of Neurology, Yale School of Medicine, Yale University, New Haven, CT, United States; ^2^Center for Neuroepidemiology and Clinical Neurological Research, Yale School of Medicine, Yale University, New Haven, CT, United States; ^3^Department of Chronic Disease Epidemiology, Yale School of Public Health, Yale University, New Haven, CT, United States; ^4^Department of Neurology and Neurotherapeutics, University of Texas Southwestern, Dallas, TX, United States

**Keywords:** essential tremor, epidemiology, genetics, endophenotype, balance, cerebellar

## Abstract

**Background:** Essential tremor (ET) cases often exhibit a range of mild cerebellar signs. Their unaffected relatives have been shown in prior studies to exhibit subtle (i.e., preclinical) disease features.

**Objective:** To quantify subtle cerebellar signs in unaffected first-degree relatives of ET cases stratified based on their tremor severity.

**Methods:** Two hundred sixty-nine first-degree relatives of ET cases, none of whom reported tremor or a diagnosis of ET, or were diagnosed with ET based on detailed neurological examination, were stratified based on total tremor score (TTS) into two groups (lower TTS vs. higher TTS) and quartiles. Changes in gait, balance, and intention tremor were quantified on neurological examination.

**Results:** Higher TTS performed worse on the tandem stance task (*p* = 0.011). When stratified into TTS quartiles, higher quartile was associated with worse performance in tandem stance (*p* = 0.011) and stance with feet together (*p* = 0.028). Similarly, intention tremor in the arms (*p* = 0.0002) and legs (*p* = 0.047) were higher in the groups with more tremor.

**Discussion:** The links between ET and the cerebellum are multiple. These data provide intriguing evidence that subtle cerebellar signs (i.e., changes in balance and intention tremor) are more prevalent among first-degree relatives of ET cases with more tremor (i.e., those who may be themselves on the pathway to developing ET). These data contribute to a better characterization of what may be an early subclinical stage of the disease.

## Introduction

Essential tremor (ET) is a chronic, progressive neurological disease whose defining clinical feature is kinetic tremor (1). Although challenges in phenotyping and other issues have hindered efforts to identify genes (2), ET is thought to be highly heritable (3); first-degree relatives of ET cases (FD-ET) are 5–10 times more likely to develop ET than are members of the general population (4). Additionally, twin studies have estimated heritability ranging from 45 to 90% (5, 6).

Recent research has focused on relatives of patients with ET, who, being predisposed for the disease, often experience subclinical tremor that is apparent on neurological examination but is too mild to meet strict diagnostic criteria (7, 8). Several studies have found significantly more tremor in first-degree relatives of ET cases (FD-ET) than in controls. Some of this tremor, although within normal bounds, is likely partially expressed ET (7, 8). This finding was even consistent across older age groups, suggesting that full disease penetrance may never be reached within a typical lifespan (7). Recent investigations have focused on several “subclinical” features and their associated impact on those without clinically defined disease (9–11). These subtle features may represent endophenotypes, or measurable clinical characteristics present in individuals with increased risk for disease. Our group previously reported that unaffected FD-ET experience diminished ability to maintain tandem stance, more near falls, and a reduction in balance confidence compared to healthy controls, suggesting a mild form of cerebellar dysfunction (11). However, research on the prevalence of other signs of subtle cerebellar dysfunction (e.g., intention tremor) in this group is lacking. To fill in this gap, we investigated the distribution of these features in a large cohort of FD-ET, none of whom fulfilled diagnostic criteria for ET. Taking our analyses one step further, we hypothesized that those with more tremor—who may be on the pathway to developing ET or are “pre-ET”—would exhibit more subtle signs of cerebellar dysfunction than those with less tremor. We *a priori* selected performance of tandem gait, stationary tandem stance, and stance with feet together; self-reported number of falls; and degree of intention tremor in the arms and legs as measures of possible cerebellar pathology. Hence, we conducted a carefully planned, limited, *a priori* comparison of the six complementary variables that reflected the same underlying issue, cerebellar dysfunction. Data that support this hypothesis would provide added support for the notion that ET arises within the context of cerebellar dysfunction (12) and would contribute to a better characterization of an early subclinical stage of the disease.

## Methods

### Introduction

FD-ET were screened for enrollment in an environmental epidemiological study of ET (May 2016 to present) at Yale University. The study and all of its protocols were approved by the Yale University institutional review board. FD-ET were relatives of ET cases who had been ascertained from study advertisements to the membership of the International Essential Tremor Foundation, membership in current ET research studies at Yale University, and the clinical practice of the Yale Movement Disorders Group. All subjects gave written informed consent in accordance with the Declaration of Helsinki.

### Screening Process

ET cases were contacted by telephone, provided informed consent, and were subjected to a telephone interview by a trained research assistant. This interview established the participant's history of tremor (including age of onset, date of diagnosis, and relative severity of tremor in each limb and the head), and surgical history (13). Each participant then completed and mailed four hand-drawn spirals, a list of current prescription medications, and an inventory of caffeine intake on the day the spirals were drawn. These materials, along with the clinical history gathered over the phone, were holistically evaluated by EDL, a senior movement disorder neurologist, using a validated scale [see definitions and examples in Louis et al. (14)].

Once a diagnosis of ET was ascertained based on these materials (i.e., moderate or greater amplitude tremor not due to another cause such as hyperthyroidism or medications), ET cases informed the investigator of all reportedly unaffected living first-degree relatives age ≥40 years. The identification and screening process for unaffected FD-ET was as follows. With permission, these family members were contacted by telephone. During this telephone call, they provided informed consent and then completed the same 12-item tremor screening questionnaire (13) as their affected relative, and were asked about a prior diagnosis of ET. They also completed and mailed four hand-drawn spirals (two right, two left), which were rated by a senior movement disorders neurologist (EDL) using the following scale: 0, 0.5, 1, 1.5, 2, and 3 [see definitions and examples in Louis et al. (14)].

FD-ET were initially categorized as unaffected if they met each of the following criteria: (1) they did not report tremor during the 12-item telephone-administered tremor screening questionnaire (i.e., they denied tremor in each of the 12 questions) (14), (2) they had never been assigned an ET diagnosis by a treating physician, and (3) each of their four screening spirals was assigned a rating <2.0 [2 = moderate amplitude oscillations required for an ET diagnosis (15)].

### In-Person Clinical Evaluation

FD-ET were invited for an in-person clinical evaluation if initially categorized as unaffected. The evaluation was conducted by trained interviewers in enrollees' homes. Questionnaires were administered to ascertain demographic features, tremor features, medical history, number of falls in previous year, and medications. For each FD-ET, the number of additional reportedly affected first-degree relatives was defined as the genetic load. The Cumulative Illness Rating Scale (CIRS) [range = 0–42 (maximum comorbidity)] (16), a measure of medical comorbidity, was administered; this assessed the presence and severity (none = 0, mild = 1, moderate = 2, severe = 3) of comorbidity in 14 body systems. Symptoms of depression were also evaluated using the Beck Depression Scale (BDI), which includes 21 items rated on a scale of 0–3, resulting in a maximum score of 63 (17).

The in-person evaluation also included a videotaped neurological examination (18), which included a detailed assessment of postural, kinetic, intention, and rest tremors, as well as dystonia and other movement disorders (19). EDL reviewed all videotaped examinations, which were de-identified (i.e., a unique subject identification number was assigned to each enrollee based on date of enrollment) and presented in order of enrollment (which was based on the availability of the study subject rather than clinical features such as tremor severity or family history information). As such, the order of review was not linked in any way to the eventual tremor severity groupings. The severity of postural and kinetic arm tremors was rated on six examination items using a reliable rating scale (20). These six tasks were sustained arm extension, pouring water between cups, drinking water from a cup, using a spoon and bowl, finger-to-nose movements, and drawing spirals. As described (14, 21), ratings for each task were 0, 0.5, 1.0, 1.5, 2, and 3 on each side; these resulted in a total tremor score (TTS) [range = 0–36 (maximum)] (19). Intention tremor, while noted as present and rated, was not included as a component of the TTS, which, when designed, was specifically a measure of the severity of postural and kinetic tremors rather than intention tremor. Indeed, the TTS was designed in 1996, many years before intention tremor was recognized as present in some patients with ET.

The videos were pseudonymized and presented to EDL who rated the six tasks on each side. While the rating on each task may have been influenced by the rating of the preceding task, prior results from our studies indicate that performance on these six separate tasks often varies to a surprisingly high degree within individuals, and the rater is aware of this high intraindividual variability and, hence, primed to treat each rating independently of others in order to capture this variability (22).

Gait and balance were assessed during videotaping in three ways. First, FD-ET were asked to complete a 10-step tandem gait task along an imaginary straight line on the ground, with the examiner ensuring their feet were touching and directly in front of each other. The number of steps off the line was noted by EDL upon review (11). Second, FD-ET were asked to hold a stationary tandem stance with one foot directly in front of and touching the other (10 s), and third, to stand with both feet side-by-side and touching at the ankles (10 s). EDL recorded the number of seconds each stance was able to be held without stepping to the side or grabbing for support (11).

The finger–nose–finger maneuver included 10 repetitions per arm (23–25). Intention tremor in each arm was defined as present when tremor amplitude increased during visually guided movements toward the target (23–25). We excluded position-specific tremor or postural tremor at the end of movement (23–25). Intention tremor was rated (EDL) in the terminal period of the finger–nose–finger test: 0 (no intention tremor), 0.5 (probable intention tremor), and 1 (definite intention tremor) (23–25). The intention tremor score (both arms combined) ranged from 0 to 2 (23–25).

To establish the degree of leg intention tremor, FD-ET were asked, while seated, to raise their foot from the ground to reach the target (a tongue blade) and touch it with their big toe. The tongue blade was placed at least 16 in from the ground level. This maneuver was repeated 10 times and was rated in the same manner as the upper limb (range = 0–2) (26).

FD-ET were re-evaluated for a potential ET diagnosis based on a review of questionnaires and videotaped neurological examination data. Diagnoses of ET were assigned based on published diagnostic criteria [moderate or greater amplitude kinetic tremor during three or more activities or a head tremor in the absence of Parkinson's Disease (PD) or another known cause (e.g., medication-induced tremor, tremor from hyperthyroidism)] (11, 15, 18).

### Final Sample

Of an initial sample of 432 FD-ET, 345 were categorized as unaffected based on the initial phone screening and evaluated in-person. Of these 345, 63 were excluded due to movements observed in the videotaped neurological examination (47 ET, 12 dystonia, and 4 ET and dystonia). Of the remaining 282, 9 were excluded due to an incomplete neurological examination preventing calculation of the TTS and 4 due to a young age that was skewing the sample. The final sample therefore consisted of 269 FD-ET with TTS ranging from 0 to 14 out of 36. We previously reported imbalance in 190 of these FD-ET compared to age-matched control subjects (11).

For the main analysis, the sample was simply stratified into two groups, “higher TTS” (HTTS) and “lower TTS” (LTTS), cutoff at the median of 6.0 out of 36. While this approach was simple and it maximized the sample size in each group (i.e., one-half of the sample), it also forced each data point into only two categories and therefore did not capture the full extent of the variance in the data (e.g., a slightly high and a very high value were both assigned to the same group), and it did not allow us to assess trends in data (e.g., the possibility that values in group 1 > group 2 > group 3 > group 4). To complement these simple analyses and address the limitations of that approach, we also performed a quartiles approach. Thus, the sample was further stratified into quartiles based on TTS. For some strata, the number of subjects was unequal because there were numerous subjects at the split point. For example, numerous subjects had a TTS = 6, and these were incorporated into the HTTS group. Final decision regarding the placement/classification of data on such cases was made homogeneously and uniformly for all individuals with that data value, without knowledge of other data items (e.g., demographic features or cerebellar signs) and with a sole goal of creating groups of as uniform size as possible.

### Statistical Analyses

All data were tested for normalcy using a Kolmogorov–Smirnov test, and non-parametric approaches were used if data were not normally distributed. HTTS and LTTS were compared with respect to demographic and clinical features (Table 1). Non-parametric tests (e.g., Wilcoxon rank-sum test) were used to compare the two groups (Table 1). For variables representing frequencies, *X*^2^ or Fisher's exact tests were used (Table 1).

**Table 1:** Demographic and clinical features of LTTS vs. HTTS.

	**LTTS (*n* = 118)**	**HTTS (*n* = 151)**	***p-*value**
Current age in years	55.7 ± 8.1 (55, 11.8)	57.6 ± 9.7 (57, 13)	0.14^a^
Female gender	89 (75.4)	94 (62.3)	0.03^b^
European ancestry	113 (95.8)	147 (97.4)	0.44^b^
English language	116 (98.3)	148 (98.0)	0.33^b^
Current cigarette smoker	2 (1.7)	6 (3.3)	0.47^b^
Number of prescription medications with potential to affect balance	0: 105 (89.0)	0: 137 (90.7)	0.23^c^
	1: 12 (10.2)	1: 11 (7.3)	
	2: 0 (0.0)	2: 3 (2.0)	
	3: 1 (0.01)	3: 0 (0.0)	
Taking a medication with potential to affect balance	13 (11.0)	14 (9.3)	0.79^b^
CIRS score	3.8 ± 2.9 (4, 5)	4.5 ± 3.7 (4, 4.3)	0.27^a^
BDI	4.6 ± 4.8 (3, 4)	4.4 ± 4.0 (3, 5)	0.98^a^
Genetic load	3.4 ± 1.0 (3, 0)	3.4 ± 0.8 (3, 1)	0.63^a^

*For continuous variables: mean ± standard deviation (median, interquartile range); for frequencies: count (percentage of total)*.

a Two-sided Wilcoxon rank-sum test

b X^2^ test

c Fisher's exact test

*BDI, Beck's Depression Inventory; CIRS, Cumulative Illness Rating Scale; HTTS, higher total tremor score; LTTS, lower total tremor score*.

Data on prescription medications were used to create a new variable (number of prescription medicines with potential to affect balance), which provided a count of current medications that could directly affect gait and balance [e.g., sedating medications and psychoactive medications associated with balance problems (e.g., anticonvulsants)] (11).

To assess whether a group (LTTS vs. HTTS) was associated with subtle cerebellar features, we compared performance on a tandem gait task, ability to maintain stationary tandem stance and stance with feet together, number of self-reported falls in the previous year, and intention tremor in the arms and legs between LTTS and HTTS (Table 2). Because the dependent variables were not normally distributed, we used two-tailed Wilcoxon rank-sum tests to compare means.

**Table 2:** Signs of cerebellar function in LTTS vs. HTTS.

	**LTTS (*n* = 118)**	**HTTS (*n =* 151)**	***p-*value^**a**^**
TTS^1^	3.63 ± 1.39 (4, 0–5)	7.97 ± 1.78 (8, 6–15)	<0.001^**^
Tandem gait (number of steps off straight line)^2^	0.54 ± 0.89 (0, 1)	1.16 ± 2.13 (0, 1)	0.29
Stationary tandem stance (number of seconds without falling to the side)	9.29 ± 2.18 (10, 0)	8.46 ± 3.15 (10, 0)	0.011^*^
Stance with feet together (number of seconds without falling to the side)	10.00 ± 0.00 (10, 0)	9.8 ± 1.40 (10, 0)	0.12
Number of falls reported in the last year	0.42 ± 1.30 (0, 0)	0.39 ± 0.87 (0, 0)	0.73
Arm intention tremor score (0–2)	0.23 ± 0.37 (0, 0.5)	0.41 ± 0.46 (0.5, 1)	0.00047^**^
Leg intention tremor score (0–2)	0.21 ± 0.44 (0, 0)	0.33 ± 0.53 (0, 1)	0.044^*^

1 For TTS: mean ± standard deviation (median, range)

2 For other variables: mean ± standard deviation (median, interquartile range)

a Two-sided Wilcoxon rank-sum test

* p < 0.05;

** p < 0.01

*LTTS, lower total tremor score; HTTS, higher total tremor score; TTS, total tremor score*.

We then further separated FD-ET into quartiles (Table 3) based on TTS. Jonckheere–Terpstra tests, which are non-parametric and account for overall trend across the quartiles, were employed to generate *p-*values for these comparisons. Because of ties in the data and the large sample size, the exact Jonckheere–Terpstra test could not be used; *p-*values were instead calculated using the permutation version of the test, with the number of permutations set to 10,000 (27). All analyses were accomplished in R 3.5.2 (Windows) using the dplyr, stats, and clinfun packages (28–30).

**Table 3:** Signs of cerebellar dysfunction stratified by TTS quartiles.

	**TTS Q1 (*n* = 78)**	**TTS Q2 (*n* = 76)**	**TTS Q3 (*n* = 64)**	**TTS Q4 (*n* = 51)**	***p*-value**
TTS^1^	2.92 ± 1.21 (3, 0–4)	5.47 ± 0.50 (5, 5–6)	7.45 ± 0.51 (7, 7–8)	10.0 ± 1.30 (10, 9–15)	<0.002^**^^a^
Age^2^	56.19 ± 7.53 (55, 10)	55.80 ± 9.52 (56, 12)	55.34 ± 9.54 (54, 12.2)	60.94 ± 9.02 (61, 11)	0.050^a^
Female gender	62 (79.5)	52 (68.4)	39 (60.9)	30 (58.8)	0.042^*^^b^
Number of prescription medications with potential to affect balance	0: 70 (89.7)	0: 70 (92.1)	0: 59 (92.2)	0: 43 (84.3)	0.22^c^
	1: 7 (9.0)	1: 6 (7.9)	1: 5 (7.8)	1: 5 (9.8)	
	2: 0 (0.0)	2: 0 (0.0)	2: 0 (0.0)	2: 3 (5.9)	
	4: 1 (1.3)	4: 0 (0.0)	4: 0 (0.0)	4: 0 (0.0)	
Tandem gait (number of steps off straight line)	0.53 ± 0.83 (0, 1)	0.82 ± 1.50 (0, 1)	0.79 ± 2.07 (0, 0)	1.67 ± 2.29 (1, 3)	0.12^a^
Stationary tandem stance (number of seconds without falling to the side)	9.34 ± 2.13 (10, 0)	8.73 ± 2.87 (10, 0)	9.12 ± 2.44 (10, 0)	7.85 ± 3.61 (10, 4)	0.011^*^^a^
Stance with feet together (number of seconds without falling to the side)	10.00 ± 0.00 (10, 0)	10.00 ± 0.00 (10, 0)	9.84 ± 1.18 (10, 0)	9.58 ± 2.02 (10, 0)	0.028^*^^a^
Number of falls reported in the last year	0.46 ± 1.53 (0, 0)	0.25 ± 0.55 (0, 0)	0.50 ± 1.13 (0, 0.5)	0.39 ± 0.70 (0, 1)	0.31^a^
Arm intention tremor score (0–2)	0.18 ± 0.32 (0, 0.5)	0.34 ± 0.42 (0, 0.5)	0.39 ± 0.44 (0, 1)	0.47 ± 0.51 (0.5, 1)	0.0002^**^^a^
Leg intention tremor score (0–2)	0.26 ± 0.50 (0, 0)	0.16 ± 0.33 (0, 0)	0.35 ± 0.58 (0, 1)	0.39 ± 0.55 (0, 1)	0.047^*^^a^

1 For TTS: mean ± standard deviation (median, range)

2 For other: mean ± standard deviation [median, interquartile range] or count (percentage)

a Two-sided Jonckheere–Terpstra test computed with 10,000 permutations

b X^2^ test

c Fisher's exact test

* p < 0.05

** p < 0.01

*Q1–Q4, quartiles one through four; TTS, total tremor score*.

To assess the effects of age and gender as potential confounding factors, we performed stratified analyses based on these variables (i.e., comparing each of the subtle cerebellar features in only male individuals across tremor severity groups and then doing the same for female individuals, and doing the same for higher age vs. lower age groups). In these analyses, which were underpowered, we qualitatively assessed whether the differences observed in the main analyses persisted.

In Discussion, we provide a detailed treatment of our reasons for not adjusting for multiple comparisons; a detailed discussion of these issues may also be found in other sources (31–33).

**Figure 1:**
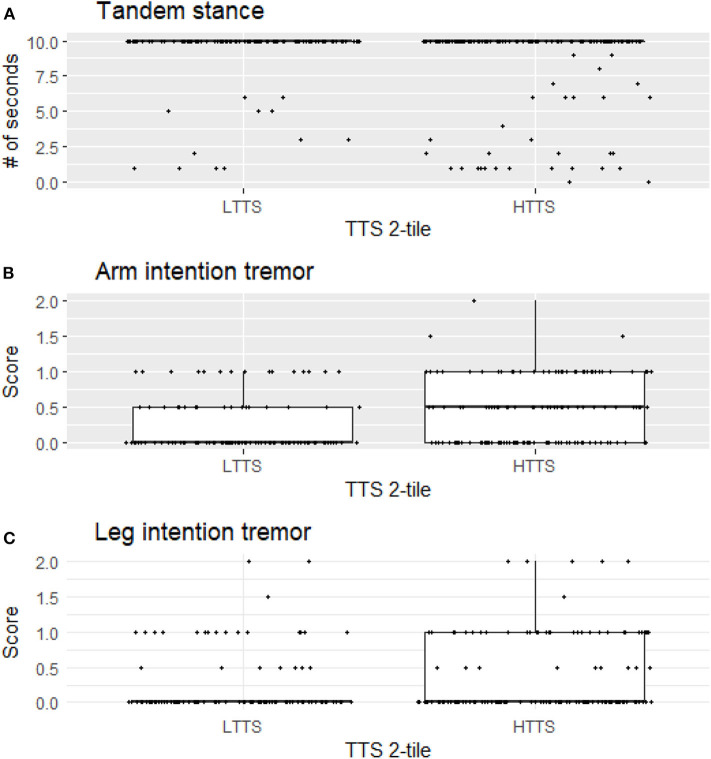
Stationary tandem stance **(A)**, arm intention tremor score **(B)**, and leg intention tremor **(C)** score differ significantly between LTTS and HTTS. LTTS, lower total tremor score; HTTS, higher total tremor score; TTS, total tremor score.

**Figure 2:**
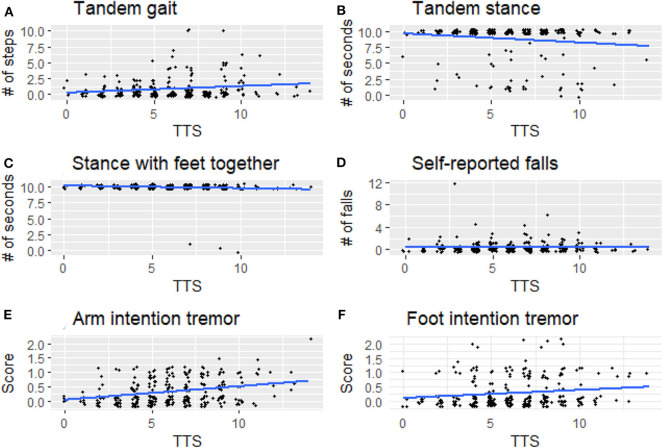
Signs of cerebellar dysfunction [**(A)** tandem gait, **(B)** tandem stance, **(C)** stance with feet together, **(D)** self-reported falls, **(E)** arm intention tremor, **(F)** foot intention tremor] plotted against TTS. TTS, total tremor score.

For illustrative purposes, we generated boxplots of the three variables that were significant in our analyses between LTTS and HTTS—stationary tandem stance, arm intention tremor, and leg intention tremor (Figure 1). In addition, we generated scatterplots of the six dependent variables vs. TTS as a continuous variable, with a trend line generated by linear regression (Figure 2). All plots were produced in R using the ggplot2 package (34).

## Results

HTTS and LTTS were similar in nearly all demographic and clinical features, including age, ancestry, language, smoking habits, CIRS score, and depressive symptoms (Table 1). The two groups were similar with respect to genetic load but differed slightly by gender. Groups were comparable with respect to the number of prescription medications that could affect balance. A visualization of scores on each of the 12 tasks in HTTS and LTTS is presented in Supplementary Figure 1. FD-ET were then stratified into TTS quartiles; each quartile was subsequently compared with respect to age, gender, and number of prescription medications that could affect balance (Table 3). Quartiles differed significantly in age (*p* = 0.050) and gender (*p* = 0.042). The quartile groups did not differ significantly in terms of the number of balance-affecting medications (*p* = 0.22). As a result of the demographic differences across groups, stratified analyses were conducted to qualitatively measure the impact of these covariates on our results (see below).

HTTS performed worse on tandem gait, missing an average of double the number of steps compared to LTTS (1.16 vs. 0.54, Table 2); however, this difference was not significant (*p* = 0.29). When FD-ET were stratified into TTS quartiles, similarly, each increase in quartile was associated with an overall increase in number of steps off the straight line (means = 0.53, 0.82, 0.79, 1.67, *p* = 0.12, Table 3).

HTTS showed significantly reduced ability to hold stationary tandem stance, averaging only 8.46 s compared to 9.29 s in LTTS (*p* = 0.011, Table 2, Figure 1A). This difference was also seen in the quartile analysis, with the quartile with the highest TTS holding the stance for ~1.5 s less than the lowest quartile (7.85 s vs. 9.34 s, *p* = 0.011, Table 3).

HTTS performed slightly worse with respect to stance with feet together, although the difference failed to reach statistical significance (*p* = 0.12, Table 2). When FD-ET were stratified into TTS quartiles, with increases in quartile, there was a marked decline in the number of seconds without falling to the side (means = 10.0, 10.0, 9.84, 9.58 s); this difference was significant (*p* = 0.028, Table 3).

Groups were also compared in terms of self-reported number of falls (Tables 2 and 3). There were no differences.

Arm intention tremor score was significantly greater in HTTS than LTTS, with the score being, on average, close to double (*p* = 0.00047, Table 2, Figure 1B). The TTS quartile analysis revealed an increase in arm intention tremor score (means = 0.18, 0.34, 0.39, 0.47, *p* = 0.0002, Table 3). Leg intention score was ~50% greater in HTTS than LTTS (*p* = 0.044, Table 2, Figure 1C). The TTS quartile analysis revealed an overall increase in leg intention tremor, although the increase was not consistent over all groups (means = 0.26, 0.16, 0.35, 0.39), with the score in the highest quartile over 1.5 times that of the lowest quartile (*p* = 0.047, Table 3). Data on each of the six signs of cerebellar function were also plotted against TTS (Figure 2).

To assess the effects of age and gender as potential confounding factors, we performed stratified analyses based on these variables (i.e., comparing each of the subtle cerebellar features in only male individuals across tremor severity groups and then doing the same for female individuals, and doing the same for higher age vs. lower age groups). In these analyses, which were underpowered, we qualitatively assessed whether the differences observed in the main analyses persisted. All differences observed in the main analyses persisted. For example, for tandem stance, values were 9.50 vs. 8.18 s (LTTS vs. HTTS) in male individuals and 9.22 vs. 8.63 s (LTTS vs. HTTS) in female individuals.

## Discussion

We analyzed data on 269 unaffected FD-ET cases and stratified them into higher or lower tremor groups based on degree of tremor exhibited on a detailed videotaped neurological examination, as assessed by a senior movement disorders neurologist. We then further stratified this cohort into quartiles based on tremor. We took great care to exclude any FD-ET who met published diagnostic criteria for ET or who exhibited any signs of other movement disorders, such as dystonia or PD, which could have confounded these results. In this carefully phenotyped sample, we found that HTTS had significantly impaired performance in tandem stance, had marginally impaired performance in stance with feet together, and exhibited significantly more intention tremor in the arms and legs. In the quartile analysis, we found that increasing quartile group was significantly associated with worse performance on tandem stance and stance with feet together, as well as with more intention tremor in the arms and legs.

We previously found that FD-ET cases reported significantly more near falls and lower balance confidence than age-matched controls (11). Taking those analyses further, we have now stratified FD-ET into “lower tremor” and “higher tremor” groups and found no significant difference in these measures between the two groups. In other words, the presence of these additional cerebellar signs does not appear to predispose the HTTS group to more falls. Thus, it is unlikely that the HTTS would experience any noticeable impairment in daily functioning.

None of these FD-ET were diagnosed with ET; indeed, any FD-ET with ET were carefully removed from this sample. Even in the HTTS group, the mean TTS was only 7.97 out of 36. A score of 8 equates to a score of 1 (mild tremor) or less on each of the 12 items rated. By comparison, in prior studies of ET cases ascertained from the population, mean TTS was 17.8–19.8 (4, 35), and in genetic and clinic-based samples, mean TTS tends to be even higher (>20) (36). A study of “borderline ET cases” (i.e., FD-ET with borderline clinical findings who did not meet strict criteria for ET) revealed a mean ± standard deviation TTS of 11.4 ± 2.6, in comparison with 7.3 ± 1.8 in normal individuals (37). Hence, by all measures across other studies, our FD-ET would have been classified as normal.

It is of value to consider our observations in their mechanistic context. Each of the assessed clinical biomarkers is influenced by cerebellar function. The most likely mechanism for the observed differences is that there is subtle cerebellar dysfunction in one group relative to the other. More specifically, from a mechanistic vantage point, these data suggest that cerebellar function is slightly, but measurably, impaired in healthy individuals with a family history of ET, and that those with more tremor exhibited an associated increase in such signs. These data lend support to the hypothesis that ET arises within the context of dysfunction of the cerebellum (23).

Although correcting for multiple comparisons is sometimes appropriate, in several situations, it is not. A discussion of several of these situations may be found in Rothman (31), Saville (32), and “When not to correct for multiple comparisons” (33). First, each of the six variables essentially measured the same underlying issue, cerebellar dysfunction, and hence were complementary and related rather than true independent entities. If the variables had really been independent, then one could have made a case for correcting for multiple comparisons, but they were merely expressions or measures of the same underlying entity. Second, the six outcome variables were selected *a priori* based on our overall hypothesis that cerebellar dysfunction is associated with increased tremor in FD-ET. This is what statisticians refer to as *planned comparison*. Numerous other variables could have been selected (e.g., a host of demographic factors, a broad array of examination features aside from the limited number of cerebellar features we focused on, numerous other clinical measures of comorbidity), but we conducted a carefully planned, limited, *a priori* comparison rather than haphazard, unplanned, multivariable, *post hoc* comparison. The issue of planned comparisons is controversial, and there are arguments in favor of forgoing multiple comparisons and those that still recommend such corrections, indicating that a rigid approach is not warranted (38) *cf*. (39). In lieu of making corrections for multiple comparisons, some authorities recommend reporting effect sizes to “let readers use their own judgment about the relative weight of the conclusions” (39), as we have done. Third, our results are not likely due to chance. With a value of alpha set at 0.05, one would expect to find statistical significance for 5% (i.e., 1 in 20) of tested hypotheses due to chance. We observed statistical significance in three of six (50.0%) of our findings in the two-tile (LTTS vs. HTTS) analysis, and four of six (66.7%) of our findings in the quartile analysis. Because these percentages far exceed the value 5%, and because most were significant across both types of analyses, it is likely that most of the observed significance was not due to chance.

Several factors limit the interpretation of these data. First, all types of tremor were evaluated clinically, not electrophysiologically, which would have aided in the precision of our measurements. Second, gait parameters were quantified visually, as opposed to through the use of computerized instruments; these would have been infeasible to transport into the field. We propose future studies to directly assess physiological mechanisms, for instance employing eye-motion tracking or machine-based gait testing. Third, controls (participants with no family history of ET) were not included in this analysis but would have added an additional dimension to these analyses. We did not include controls because the hypothesis that these analyses were designed to test required that we focus on variation in six outcome variables as these related to another variable, mild tremor, *specifically in relatives of ET cases*. It is furthermore important to note that the observed associations do not represent normal variation in data; they represent the variation of one element of data (subtle cerebellar signs) with respect to another data element (tremor severity) in an at-risk population—relatives of ET cases rather than in the general population. Although in many instances statistically significant, any difference between groups was subtle and likely subclinical, as our study subjects, by intention and design, comprised *unaffected individuals* who did not have a neurological disease, as our hypothesis was to assess subtle signs of cerebellar dysfunction in *unaffected* relatives. As a result, this analysis did not lend itself to a discussion of sensitivity and specificity—in other words, we are not suggesting that the presence or absence of the assessed subtle cerebellar signs be used clinically to differentiate the two groups as our goal was not to develop a diagnostic differentiator. Indeed, the graphical data (Figures 1, 2) show a high degree of overlap between low- and high-tremor groups. Finally, the data are cross-sectional rather than longitudinal. Hence, we do not have data on the conversion of these individuals to incident ET. Those data, however, would be of great interest and value.

This study also had a number of strengths. To our knowledge, it is the only study to compare all of these clinical features in FD-ET and the only one in which they were further stratified by tremor severity. Second, all FD-ET were evaluated prospectively using a standardized research protocol. Third, the large sample size, of more than 250, allowed for well-powered analyses. Fourth, we considered the potential effects of numerous confounding factors, including age, gender, and influence of prescription medications on balance.

In summary, we examined performance on various gait and balance tasks, number of self-reported falls, and degree of intention tremor in 269 healthy FD-ET. Relatives with increased tremor exhibited significantly worse performance in tandem stance and stance with feet together, and more intention tremor in the arms and legs. Because the above features are often linked to cerebellar dysfunction, these data provide further support for the notion that ET arises within the context of cerebellar dysfunction and pathology.

## Data Availability Statement

The raw data supporting the conclusions of this article will be made available by the authors, without undue reservation.

## Ethics Statement

The studies involving human participants were reviewed and approved by Yale University Institutional Review Boards. The patients/participants provided their written informed consent to participate in this study.

## Author Contributions

EH acquisition of data, analysis and interpretation of data, drafting/editing manuscript, and final approval of work. RH, HD, DV, and GL acquisition of data, drafting/editing manuscript, and final approval of work. EL conception and design of study, analysis and interpretation of data, drafting/editing manuscript, and final approval of work. All authors contributed to the article and approved the submitted version.

## Conflict of Interest

The authors declare that the research was conducted in the absence of any commercial or financial relationships that could be construed as a potential conflict of interest.
