# Supplementary material for: Subtle Cerebellar Features in Relatives of Essential Tremor Cases
Source: Front Neurol. 2020 Jul 17;11:605. doi: 10.3389/fneur.2020.00605 (PMC7379149; doi:10.3389/fneur.2020.00605)
Supplement: Supplementary file 1 [file Data_Sheet_1.docx]

**Supplementary Figure 1:** Comparison of LTTS and HTTS in each rated task on the videotaped neurological examination


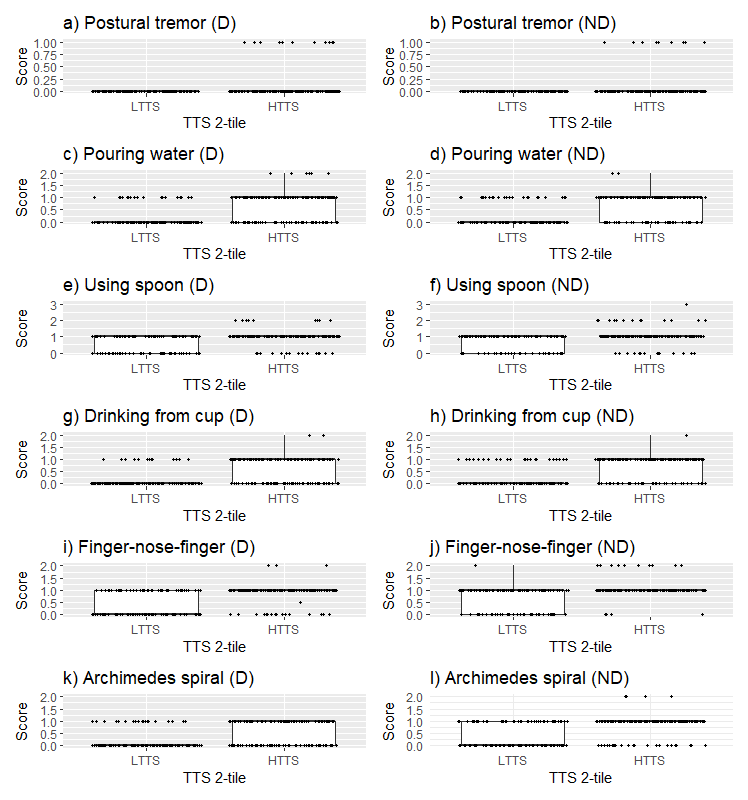


Abbreviations used: D (Dominant arm), ND (Non-dominant arm), LTTS (lower total tremor score), HTTS (higher total tremor score), TTS (total tremor score)
